# Filament Breakage Monitoring in Fused Deposition Modeling Using Acoustic Emission Technique

**DOI:** 10.3390/s18030749

**Published:** 2018-03-01

**Authors:** Zhensheng Yang, Li Jin, Youruiling Yan, Yiming Mei

**Affiliations:** 1 School of Logistics Engineering, Shanghai Maritime University, Shanghai 201306, China; gege1964713@126.com (L.J.); oldfacehhh@126.com (Y.Y.); 2 The State Key Laboratory of Fluid Power Transmission and Control, College of Mechanical Engineering, Zhejiang University, Hangzhou 310027, China; meiyiming2012@163.com

**Keywords:** additive manufacturing, fused deposition modeling, filament breakage, acoustic emission, monitoring

## Abstract

Polymers are being used in a wide range of Additive Manufacturing (AM) applications and have been shown to have tremendous potential for producing complex, individually customized parts. In order to improve part quality, it is essential to identify and monitor the process malfunctions of polymer-based AM. The present work endeavored to develop an alternative method for filament breakage identification in the Fused Deposition Modeling (FDM) AM process. The Acoustic Emission (AE) technique was applied due to the fact that it had the capability of detecting bursting and weak signals, especially from complex background noises. The mechanism of filament breakage was depicted thoroughly. The relationship between the process parameters and critical feed rate was obtained. In addition, the framework of filament breakage detection based on the instantaneous skewness and relative similarity of the AE raw waveform was illustrated. Afterwards, we conducted several filament breakage tests to validate their feasibility and effectiveness. Results revealed that the breakage could be successfully identified. Achievements of the present work could be further used to develop a comprehensive in situ FDM monitoring system with moderate cost.

## 1. Introduction

The past three decades have witnessed the rapid growth of Additive Manufacturing (AM) technologies. Especially during the last five years, AM has gained widespread attention not only from the academic community, but also the public. Companies across the globe are using AM to reduce time-to-market, improve product quality and reduce the cost to manufacture products. In the industrial sector, polymer-based AM techniques are being used in a wide range of part applications including automotive, aerospace and medical devices [1]. The most widely-used and rapidly-growing AM technologies are extrusion deposition processes such as Fused Deposition Modeling (FDM), Fused Filament Fabrication (FFF) and Melt Extrusion Manufacturing (MEM) [2]. While the use of AM has been growing, numerous challenges impede its more widespread adoption and commercialization [3]. One of the most urgent issues is the in situ monitoring of the AM process.

In order to manufacture high quality parts using AM technology, it is essential to be able to monitor the critical process parameters and malfunctions as a part is being manufactured. Process monitoring is essential to detect defects and provide feedback for process control, which is key to further understanding AM processes, improving process efficiency and quality and producing parts with desired qualities. A comprehensive review of the process monitoring of metal additive manufacturing such as the Stereo Lithography Apparatus (SLA) and Selective Laser Melting (SLM) can be found in [4,5]. This work focused on the process monitoring of polymer-based AM, e.g., fused deposition modeling, in particular.

The process monitoring of the FDM process is challenging. The FDM process is known to be exceedingly sensitive to variations in processing conditions/settings. In situ measurement is needed to monitor various process parameters such as temperature, feed rate and material properties. Defects and malfunctions should also be identified in time to provide feedback for process control. Research efforts on this scientific issue can be divided into three fields, i.e., (1) modeling and characterization of the AM process, structure and thermal properties, (2) measurement for part quality and (3) monitoring technology. The characterization of the structure and thermal properties endeavors to understand the molecular structure, fiber orientation, thermal properties, stress and strain properties [3]. In order to overcome the limited techniques in FDM machines, Yedige et al. built a physics-based dynamic model for nozzle clog monitoring. Based on the model, it was found that the mounting of a liquefier block in an FDM extruder can be used to place a vibration sensor to monitor nozzle clogging [6]. Zhang et al. investigated the influence of process conditions on the temperature variation in the FDM process, which provides insights into understanding the FDM process from the perspective of energy balance [7]. The monitoring of part quality is important for further deployment of FDM AM technologies. The influence of design parameters on part surface roughness and dimensional accuracy was thoroughly reviewed in [8]. Boschetto et al. proposed a prediction model using a neural network to estimate the surface roughness of FDM parts based on process parameters [9]. Further studies carried out by Vahabli and Rahmati can be found in [10]. Mohammad and Jain studied surface roughness prediction based on the monitoring of the build edge profile of each deposited layer, such as the perimeter, raster and the combination of both layer deposition patterns [11].

The monitoring of FDM using various sensors plays an important role in understanding the technology and building control systems for the manufacturing process. Rao et al. used a heterogeneous sensor array including a filament feed speed sensor, borescope camera, MEMS accelerometer, thermocouple and IR temperature sensor to identify failure modes and detect the onset of process anomalies in the FFF process [12]. The nonparametric Bayesian Dirichlet Process (DP) mixture model and Evidence Theory (ET) were used to detect FFF process failures online, based on the experimentally-acquired sensor array data. Fang et al. proposed a vision-based monitoring system of ceramics’ fused deposition, in which the optical image of each layer was captured and compared with the ideal layer morphology using machine vision techniques [13]. Kim et al. studied a methodology to detect material deposition status and solve problems like nozzle clogging and substrate deformation in FDM 3D printing, by sensing the inner pressure change of the liquefied material [14].

The present work focused on filament breakage detection in the FDM AM process. Filament breakage usually occurs when the filament is affected by humidity, or inhomogeneous filament materials sustain nonuniform pulling force. The breakage of the filament is one of the most significant process errors in FDM and may cause several malfunctions such as nozzle clogging, geometrical misalignments or manufacturing failure. However, most of the desktop FDM machines are not integrated with a breakage detection module. Mittelman and Roman used AE technique to characterize the failure mechanisms of composite materials [15,16]. The AE peak amplitude distribution skewness was used to monitor composite failure. It was noticed that the filament failure was related to the AE skewness. For industrial applications, the detection of filament breakage is realized by utilizing an optoelectronic contact switch or a mechanical contact switch, which identifies the breakage through the detection of the filament in the hole. The problem with the contact switch is that it is easy to detect whether there is a filament in the hole. However, using one single contact switch, it is difficult to determine whether the filament is static or moving. This is very important since in most circumstances, the rest of the broken filament still stays in the contact switch hole after it breaks, which means that it is incapable of detecting the breakage effectively. MakerBot Industries (New York, NY, USA) announced an extruder integrated with an encoder wheel in 2016. The wheel measures the increments of the filament’s movement to prevent under-extrusion and false filament detection, which could be used for breakage detection. However, an encoder wheel is a highly integrated component, which is impractical for most of the commercial AM machines.

To address this issue, the present work investigated the fundamental mechanism of filament breakage. An acoustic emission sensor was used for breakage identification, due to the fact that it was capable of detecting weak and bursting signals. In addition, it can be used not only in filament breakage identification, but also for other malfunctions such as clogging, material defects and part quality.

## 2. Methodology

### 2.1. Analysis of Filament Breakage in FDM

**Figure 1:**
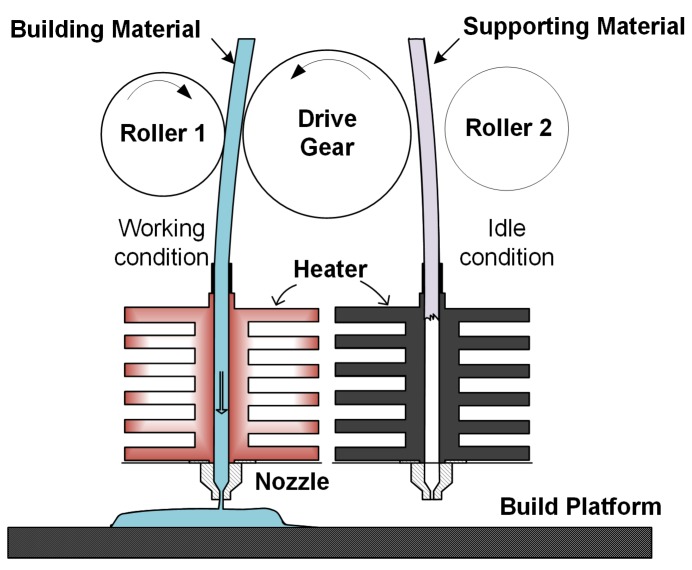
Schematic diagram of extrusion system of FDM.

Typical extrusion manufacturing machines consist of feed pinch rollers, a liquefier, a build platform and a filament feedstock component. The feedstock is generally shaped into filaments, which are made of amorphous thermoplastic polymers such as Acrylonitrile Butadiene Styrene (ABS), Polylactic Acid (PLA) and Polypropylene (PP), with a diameter of 1.75 or 3 mm. The feedstock is usually coiled inside a cartridge and pushes through the machine using a pair of pinch rollers (Figure 1). The surface of the roller is fabricated with grooved teeth, in order to create sufficient friction to grab the filament and feed it into the liquefier without slippage [17]. One of the rollers is driven by a stepper motor to move the filament through the system.

**Figure 2:**
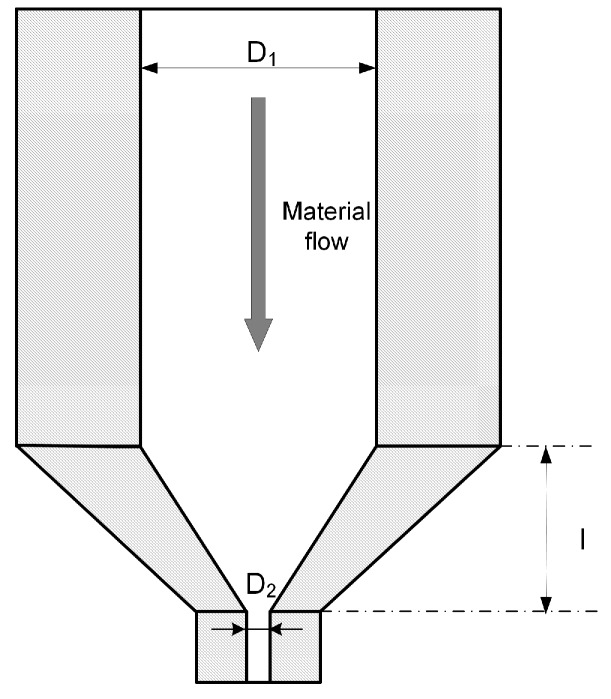
Key dimensions of the printing nozzle.

The ratio of feed to flow rates is a key factor that influences part build quality. The feed rate is controlled so as to maintain a constant volumetric flow rate of material through the print nozzle, which can be approximated as [17,18]:

(1) $$v=\frac{Q}{WH}$$

where *v* is the feed rate, *W* is the road width, *H* is the slice thickness, and *Q* is the volumetric flow rate of material from the nozzle. The volumetric flow rate *Q* is a function of the geometric parameters of the nozzle, material viscosity η and the pressure of the nozzle *P* [19]:

(2) $$Q=\frac{\pi {\left(\frac{D_{2}}{2}\right)}^{4}P}{8\eta l}$$

where $D_{2}$ is the diameter of the nozzle and *l* is the length of the nozzle’s conical shape (Figure 2).

The feed rate *v* is limited by the compression force on the liquefier side of the feed roller [8].When this feed rate reaches a critical limit, the feedstock filament can buckle. This is the most common failure mode in extrusion AM processes. Buckling can lead to the stacking of the filament between pinch rollers and the liquefier, which brings the failure of feed flow and, finally, the breakage of the filament. The critical feed rate is affected by pressure *P* placed on the filament, which can be obtained from Euler buckling analysis,

(3) $$P=\frac{\pi^{2}Ed^{2}}{16L^{2}}$$

where *E* is the elastic modulus of the filament, *d* is the diameter of the filament and *L* is the filament length from the pinch rollers to the entrance of the liquefier [20]. According to Equations (1)–(3), one can deduce the critical feed rate $v_{cr}$:

(4) $$v_{cr}=\frac{\beta Ed^{2}D_{2}^{4}}{\eta lL^{2}WH}$$

where β is the critical factor. In contrast, a lower value for the feed to flow ratio indicates extrusion of material at a faster rate than the movement of the filament. This results in thicker layers and a gradual buildup of extrudate around the nozzle, typically leading to clogs. If this condition is not changed, filament breakage will occur, and a portion of the filament will remain lodged inside the extruder [12]. The handling of clogs and filament breakage comprise troublesome processes and require disassembly of the extruder. Therefore, it is necessary to predict such catastrophic failure in advance.

### 2.2. Foundations of the Acoustic Emission Technique

Acoustic emission is defined as high-frequency stress waves generated by the rapid release of energy that occurs within a material [21]. It is the phenomenon of transient elastic wave radiation in solids that occurs when a material undergoes irreversible changes in its internal structure. Typical acoustic emission frequencies are in the range of 10 kHz–1 MHz. Crack growth, plastic deformation, phase transformation and friction can release remarkable elastic waves and can be captured through piezoelectric transducers. Ever since its discovery in the early 1950s, there has been a tremendous growth in the use of acoustic emissions in machine failure diagnosis [22,23], electric power system [24], civil engineering [25], etc. It has also been proven to be a promising technique in the emerging research field of AM process monitoring [26,27]. However, the potential of acoustic emission is underestimated in AM process monitoring. Known as a Nondestructive Testing (NDT) technique, acoustic emission sensors are capable of monitoring a great quantity of process malfunctions in AM processes. Part quality, material defects, phase transformation and clogging can be identified through only one or two acoustic emission sensors. Therefore, considering their widespread applicability, AE is a promising process monitoring technique in FDM.

### 2.3. Filament Breakage Identification Based on AE Instantaneous Skewness and Relative Similarity

In most cases, filament breakage occurred at a place close to the pinch rollers or the extruder. The current apparatus was incapable of identifying the failure efficiently. This is because the rest of the filament still remained inside the pipeline since there was no force to drag it out of the cartridge after breakage, as mentioned in Section 1. Therefore, an alternative method is required.

In the case of operations under steady state conditions, filaments were continuously grabbed by the grooved teeth on the pinch rollers and fed into the liquefier/extruder. As a result, filaments started to rub the liquefier, and the molten material was extruded from the nozzle thereafter. The extrusion of molten material could generate weak, but steady friction, along with the whole extrusion process. However, when a filament breakage occurred, there was no more filament feeding into the extruder. The compression pressure generated by the feeding of filament disappears. No more material was extruded from the nozzle. The acoustic emission should be different from the extrusion process. Such differences could be further utilized for identification.

Currently, conventional AE feature extraction approaches involve extracting information from either parametric AE signals or raw waveforms, utilizing various signal processing technology including the time domain, frequency domain or both. In most circumstances, feature extraction methods of the AE signals were mostly based on the experiences accumulated in other signal processing technologies, not the features of the AE signal itself. Parametric processing methods such as AE hits were customized for acoustic emission. However, they are highly experience-dependent, and most of the information is lost inevitably.

In the present work, an AE representation method based on the shape of the probability distribution was proposed. It is independent of the threshold or prior experiences. Features were extracted based on the representation of the raw AE’s probability distribution. For this purpose, the skewness and relative similarity of the raw AE signal were proposed to represent the differences between the two conditions.

#### 2.3.1. Instantaneous Skewness

**Figure 3:**
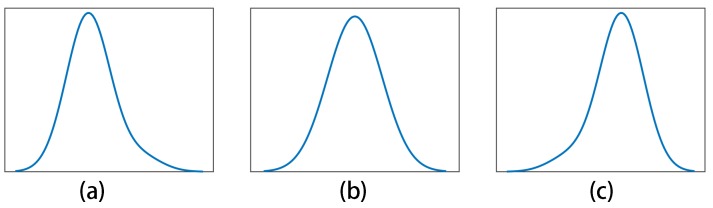
Probability distribution shape and skew: (**a**) positive skew; (**b**) zero skew; (**c**) negative skew.

Skewness γ is defined as the third standardized moment to measure the asymmetry of the probability distribution of a real-valued random variable about its mean. It is a measure of the asymmetry of the probability distribution of the data points about its mean. Zero skew means that the tails on both sides of the mean of the data points balance out overall. For a negative skew, the tail on the left side of the probability density function is longer or fatter than the right side. On the contrary, a positive skew indicates that the tail on the right side is longer or fatter than the left side (Figure 3). Generally speaking, the probability density distribution of AE is an abnormal distribution, i.e., the skewness is not zero. In addition, it is reasonable to deduce that the probability density distributions of the AE signal under different machining conditions should be different from each other. Therefore, the skewness was utilized as an indicator of the filament breakage.

In order to identify the breakage in real time, a framework named instantaneous skewness was proposed and formulated as follows.

1. Data acquisition: Collect raw acoustic emission waveforms via the Data Acquisition (DAQ) system.
2. Pre-processing: The raw waveform should be split into equilong time sections for further processing. The length of the section depends on the monitoring object. The section length should be cautiously determined because it is closely related to the temporal resolution.
3. Instantaneous skewness calculation: Calculate the instantaneous skewness $\gamma_{instant}$ based on Equation (5),
  (5) $$\gamma_{instant}=\frac{1}{\sigma_{i}^{3}N}\sum_{i=1}^{N}{\left(x-\mu_{i}\right)}^{3}$$
  where $\sigma_{i}$ is the *i*-th section’s standard deviation. *N* is the length of each section. *x* is the signal amplitude in the *i*-th section. $\mu_{i}$ is the mean of the *i*-th section.

#### 2.3.2. Relative Similarity

Instantaneous skewness is a measure of the asymmetry of the probability distribution of the AE signal. It could be used to roughly evaluate the skew variation along with time. However, it cannot represent the precise differences or similarity between two time sections. Being awareness of this, we proposed a new AE feature extraction method, namely relative similarity, to represent the differences between two AE probability distributions more precisely.

The Bhattacharyya Coefficient (BC) was introduced for this purpose. BC is a measure of the amount of overlap between two statistical samples. It can be used to determine the relative closeness of the two samples being considered. Calculating the BC involves a rudimentary form of integration of the overlap of the two samples. For discrete probability distributions, the Bhattacharyya coefficient is defined as,

(6) $$b_{c}\left(p,q\right)=\sum_{i=1}^{n}\sqrt{\left(p_{i}\cdot q_{i}\right)},$$

where, considering the samples *p* and *q*, *n* is the number of bins, and $p_{i}$, $q_{i}$ are the frequency of samples *p* and *q* in the *i*-th bin,

(7) $$p_{i}=\frac{f_{i}}{f_{n}},$$

where $f_{i}$ is the number of members of sample *p* in the *i*-th bin. $f_{n}$ is the total number of members of sample *p*.

The meaning of BC is concise and explicit. It is larger and closer to one with each bin that has members from both samples or both samples have larger overlap members, while it is smaller and closer to zero when each bin has less overlap members. In the present application, considering two AE samples, which were generated under the same condition, their skewness values were similar to each other, and the majority of the members were overlapped. As a result, the BC should be larger and closer to one. On the contrary, AE samples generated under different conditions should have less overlap members. As a result, the BC should be smaller and closer to zero.

Based on the aforementioned attribute of BC, we defined the AE discrete probability distribution relative similarity as the BC value of two AE signals. The framework of calculating AE discrete probability distribution similarity using BC is formulated as follows.

**Figure 4:**
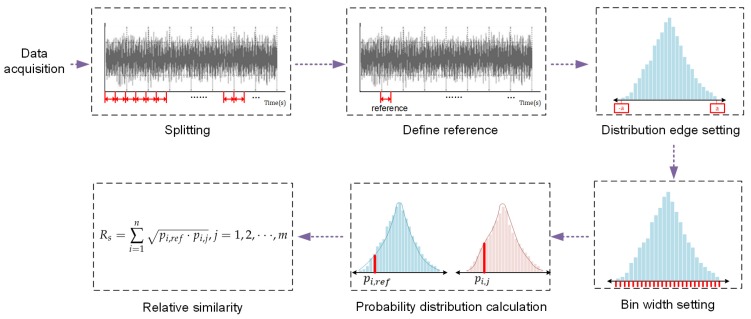
Framework of calculating AE discrete probability distribution relative similarity.

1. Data acquisition: Collect raw acoustic emission waveforms via the DAQ system.
2. Splitting: Split the raw waveform into equilong time sections.
3. Define reference distribution: A random time section should be singled out and defined as the reference. Its distribution is denoted as reference distribution $p_{ref}$. The reference distribution is usually selected from steady machining conditions.
4. Distribution edge setting: A suitable distribution edge should be assigned for probability distribution calculation. The distribution edge or boundary should cover the maximum absolute value of AE amplitude during monitoring.
5. Bin width setting: The bin width is the partition in which discrete probability distributions are calculated. The bin width should be set as close to the resolution of the DAQ system as possible, since the resolution is bound up with the signal quantity and coding.
6. Probability distribution calculation: $p_{i,ref}$ should be calculated for the *i*-th bin of reference time section. $q_{i,j}$ should be calculated for the corresponding *i*-th bin of another *j*-th section.
7. Bhattacharyya coefficient calculation: Calculate $R_{s}$ based on Equation (8), which is derived from Equation (6).
  (8) $$R_{s}=\sum_{i=1}^{n}\sqrt{p_{i,ref}\cdot p_{i,j}},j=1,2,\cdots ,m,$$
  where *m* is the number of the time sections. The framework to represent AE discrete probability distribution relative similarity using the Bhattacharyya coefficient is illustrated in Figure 4.

## 3. Experimental Setup

### 3.1. Setup of Monitoring Systems

**Figure 5:**
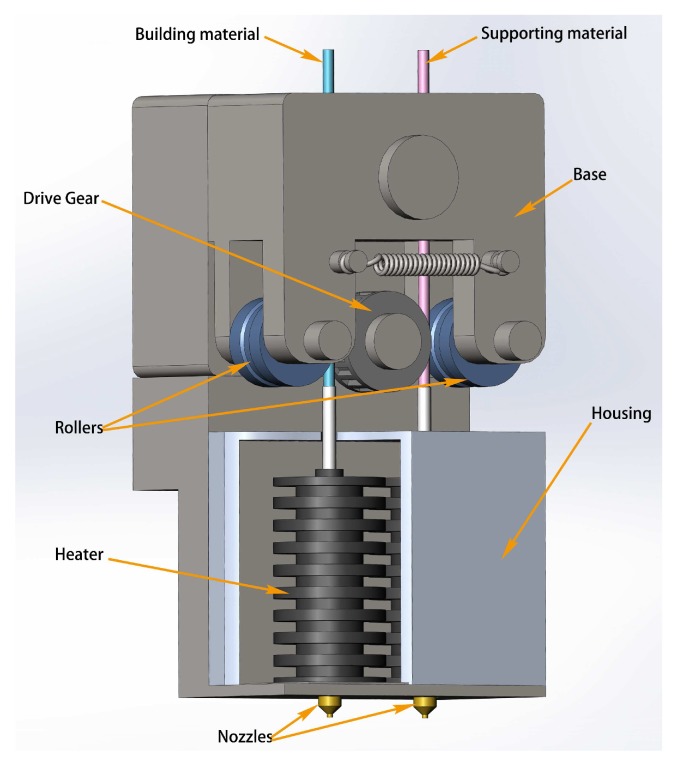
3D layout of the FDM extruder.

To verify the proposed approach for filament breakage detection via acoustic emission, a monitoring system was built. In order to detect the acoustic emission signals and eliminate noises, an optimal placement for sensor mounting is of great importance. Major acoustic emission signals during the printing process are generated from the electric motors, the movement of the printing head, the friction between the filament and pinch rollers and the extruding process. However, compared with the other two sources, the extruding generates rather weak AE signals. Thus, the sensor should be mounted as close to the extruder as possible. Since most of the extruders operate at temperatures between 50 and 200 °C and are enclosed inside a housing, there is a need to find a waveguide tightly attached to the extruder’s surface. After several trial and error tests, it is found that the housing, which is physically connected with the extruder/liquefier, has a proper surface temperature (lower than 100 °C) and could be used as an optimal waveguide (Figure 5).

**Figure 6:**
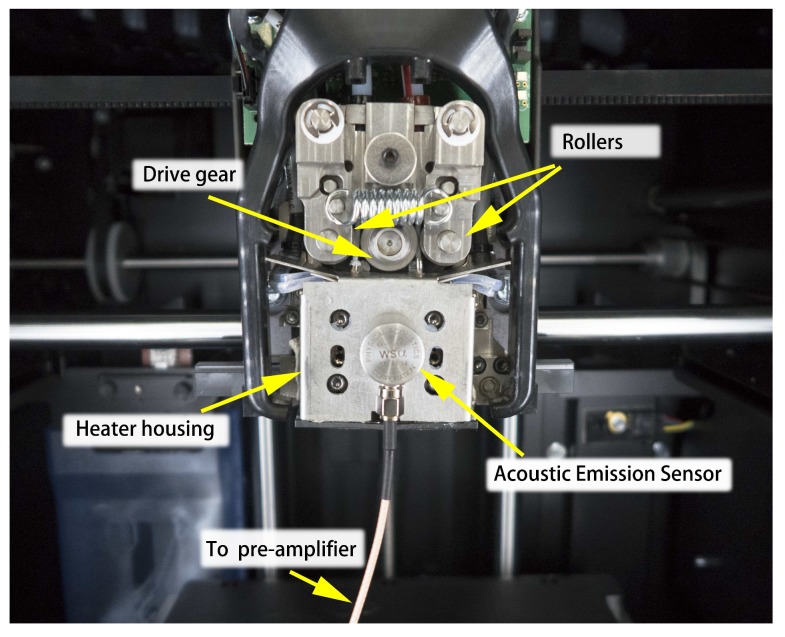
Sensor placement.

The acoustic emission instrumentation was made up of acoustic emission transducers, preamplifiers and an acoustic emission acquisition system (PCI-2, Physical Acoustic Corp., New York, NY, USA). A wide band and heat-resistant transducer (Model: WSa, Physical Acoustic Corp., New York, NY, USA) was used, with operation frequency bands ranging from 100 kHz–1000 kHz. Its working temperature ranges from −65–175 °C, which ensures a consistent level of signal collection. The transducer head was mounted on the extruder’s shell using glue (Figure 6). The interface of the two surfaces was filled with high-temperature resistant vacuum grease. The sampling frequency for the recording waveforms was 5 MHz. The waveforms were amplified with a 40-dB gain by a preamplifier.

### 3.2. Filament Breakage Test

The filament breakage monitoring systems were tested on two FDM machines (a printer from Statasys Ltd., New York, NY, USA and a printer from JG Aurora, Shenzhen, China, Table 1). Tests on the Stratasys machine, marked as Test #1, were to explore the feasibility of the monitoring system, while tests on the other machine, marked as Test #2, were to verify its universality.

Filament breakage in the two sets of tests was implemented in different ways. With regard to the exploration test on uPrint SE Plus, artificial clipping was made at a distance of 200 mm ahead of the extruder. Filament that has already been fed into the pinch roller continued moving into the extruder, while the rest of the filament remained in the cartridge due to the lack of drag force. The machine proceeded to print until all of the 200 mm of filament were fed into the extruder. The whole process was monitoring through an acoustic emission sensor attached on the surface of the extruder’s shell, as illustrated in Section 3.1.

For the universality verification tests on the JG Aurora printer, filament breakage was realized through the modulation of process parameters according to the analysis result in Section 2, in order to best mimic the actual working conditions. Based on the critical feed rate model (Equation (4)) and experimental trials, a critical factor of 0.015 was obtained, and the critical feed rate was 72.5 mm/s. Table 2 lists the key parameters. Buckling occurred 12 min after the printing started, and the filament was broken therewith. Acoustic emission signals were recorded both in the normal conditions and the filament breakage states.

## 4. Results and Discussion

### 4.1. Results

**Figure 7:**
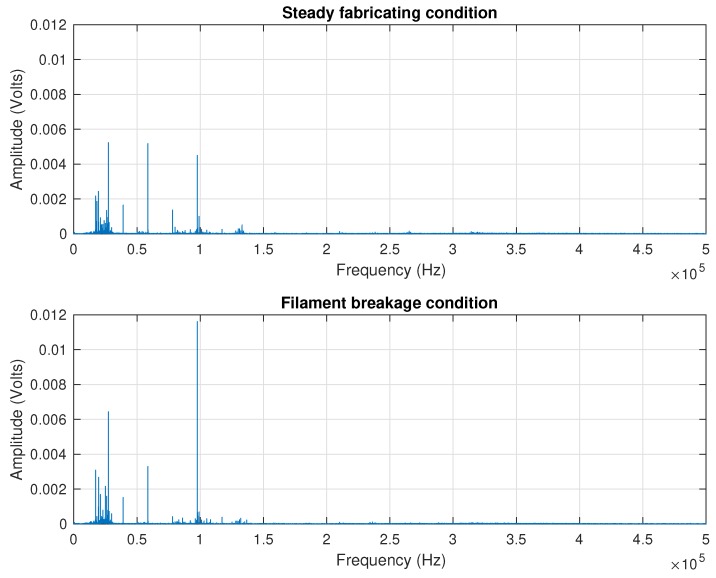
Amplitude spectrum of acoustic emission signals under different manufacturing conditions.

Raw acoustic emission signals were acquired in order to obtain abundant information about the FDM AM processes. The sampling rate of acoustic emission was as high as five mega-samples per second. It generated $1\times 10^{8}\;\mathrm{bytes}$ every 10 s ((5 $\times 10^{6}\;\mathrm{samples})\times \left(2\;\mathrm{bytes}\right)\times (10\;s$)). It occupied a huge storage space and consumed considerable computing resources at the same time. Being awareness of such a situation, spectrum analysis was performed in order to check the frequency distribution and to determine a proper sampling rate. The frequency distributions of the steady fabrication condition and breakage condition are displayed in Figure 7. Overall, the graphs were occupied by low frequency bands, both under steady fabrication and breakage conditions. To be more specific, the frequency ranged from several hertz to 150 kHz. In addition, there was a slight increase in the amplitude after breakage occurred. The frequency at 100 kHz experienced a small increase. However, it was not that appreciable. Nevertheless, Figure 7 can help us improve the sampling rate. According to the frequency distribution to the graph, a smaller sampling rate, i.e., 1 MHz, was redefined for the purpose of reducing computation resources.

Afterwards, the raw AE signals were transformed based on the proposed instantaneous skewness. The calculation of skewness using Equation (5) involves the traversal of all the datasets to obtain their mean value and variance. For the purpose of reducing computational burden, the origin moments were used for simplification as follows,

(9) $$\mu_{i}=E\left[x\right]$$

(10) $$\sigma_{i}^{2}=E\left[{\left(x-E\left[x\right]\right)}^{2}\right]=E\left[x^{2}\right]-E^{2}\left[x\right]=E\left[x^{2}\right]-\mu_{i}^{2}$$

accordingly,

(11) $$\begin{array}{c} \gamma =\frac{1}{\sigma_{i}^{3}N}\sum_{i=1}^{N}{\left(x-\mu_{i}\right)}^{3}\\ =E[{\left(\frac{x-\mu_{i}}{\sigma_{i}}\right)}^{3}]\\ =\frac{E[{\left(x-\mu_{i}\right)}^{3}]}{\sigma_{i}^{3}}\\ =\frac{E\left[x^{3}\right]-3\mu_{i}E\left[x^{2}\right]+3\mu_{i}^{2}E\left[x\right]-\mu_{i}^{3}}{\sigma_{i}^{3}}\\ =\frac{E\left[x^{3}\right]-3\mu_{i}E\left[x^{2}\right]+2\mu_{i}^{3}}{\sigma_{i}^{3}}\\ =\frac{E\left[x^{3}\right]-3E\left[x\right]E\left[x^{2}\right]+2E^{3}\left[x\right]}{{\left(E\left[x^{2}\right]-E^{2}\left[x\right]\right)}^{3/2}} \end{array}$$

On the basis of the above-mentioned equations, the moving skewness is defined using the following equation,

(12) $$\gamma_{instant}\left(n\right)=\frac{E\left[x^{3}\left(n\right)\right]-3E\left[x\left(n\right)\right]E\left[x^{2}\left(n\right)\right]+2E^{3}\left[x\left(n\right)\right]}{{\left(E\left[x^{2}\left(n\right)\right]-E^{2}\left[x\left(n\right)\right]\right)}^{3/2}}$$

where x(n) is each of the amplitude values of the *n*-th section.

**Figure 8:**
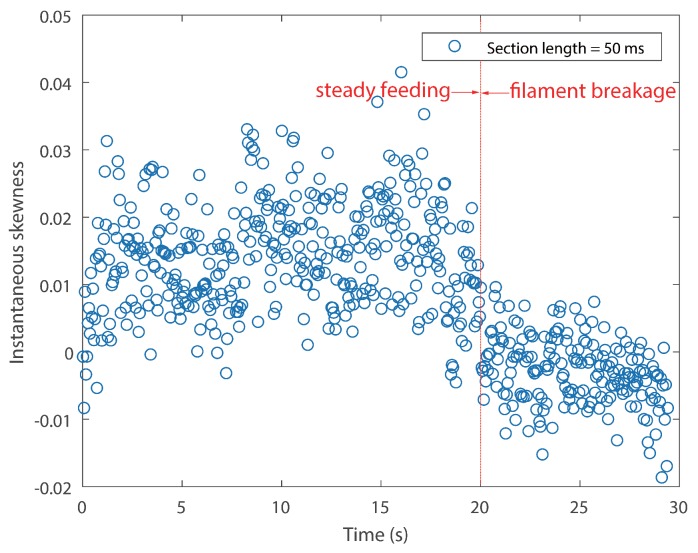
Instantaneous skewness of the AE signal for Test #1 (section length = 50 ms).

**Figure 9:**
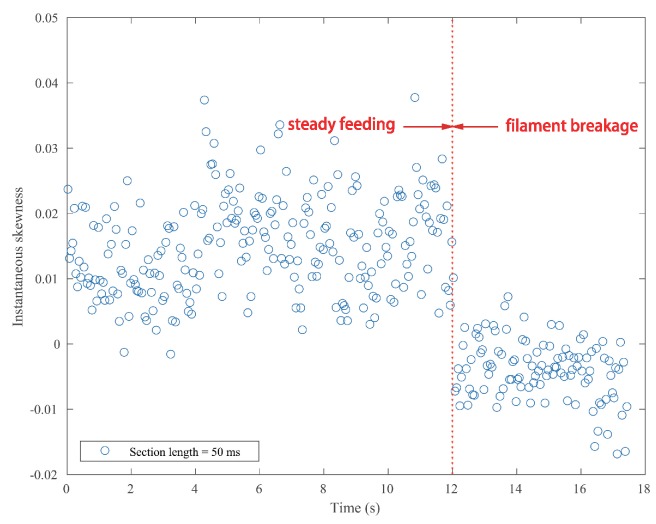
Instantaneous skewness of AE signal for Test #2 (section length = 50 ms).

In order to identify the filament breakage without delay, as well as to reduce the total computation time, an optimal signal section length *N* has to be determined. A smaller length results in larger data points, which makes the calculation of skewness time consuming. Furthermore, during the manufacturing process, the feed rate was controlled under 25 mm/s. The length of the nozzle was 59 mm. Therefore, it will take the filament approximately 2.5 s to pass through the nozzle, which means that the time of breakage alarming could be as short as 2.36 s, provided that a suitable section *N* is given. Accordingly, the length *N* is designated as 5 $\times 10^{5}$, which is 50 ms correspondingly. The result of Test #1 is displayed in Figure 8. Figure 9 shows the instantaneous skewness of Test #2.

As shown in Figure 8, the graph is roughly divided into two stages. The skewness went up and down in the first stage. However, it roughly maintained a positive level. In contrast, the skewness value crept down below zero after the filament broke. A similar tendency is also noticed in Figure 9. As mentioned above, a positive skew indicated that the mass of the distribution is concentrated on the left of the figure. The mean is being skewed to the left of a typical center of the acoustic emission signal, and vice versa. For the steady manufacturing condition, acoustic emission signals produced by the extruder were rather weak. This is because of the feeble friction between melting material and the inner wall of the extruder. As a consequence, its discrete probability density distribution was slightly right-skewed. In contrast, the rest of the broken filament, which cannot be fed into the extruder will be constantly rubbing with the pinch rollers. The friction could produce slightly higher emissions than the steady condition. The narrow differences could be magnified in their probability distribution.

**Figure 10:**
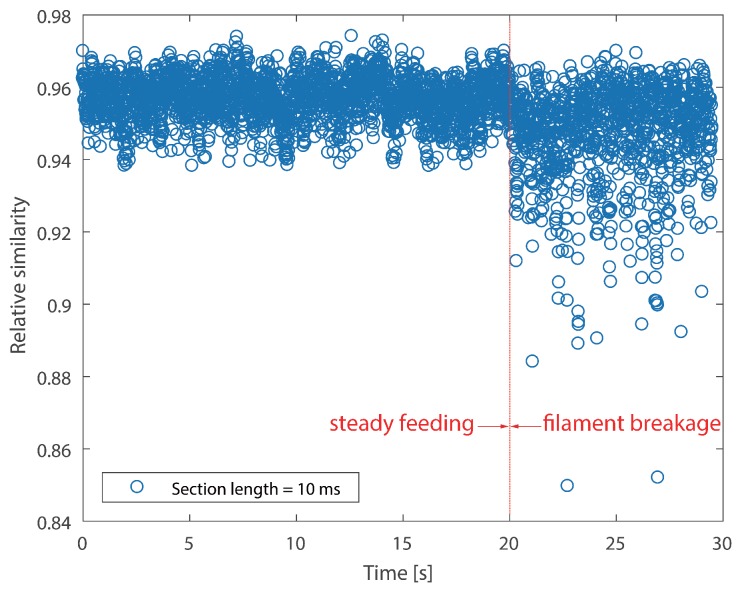
Relative similarity of the AE signal for Test #1 (section length = 10 ms).

**Figure 11:**
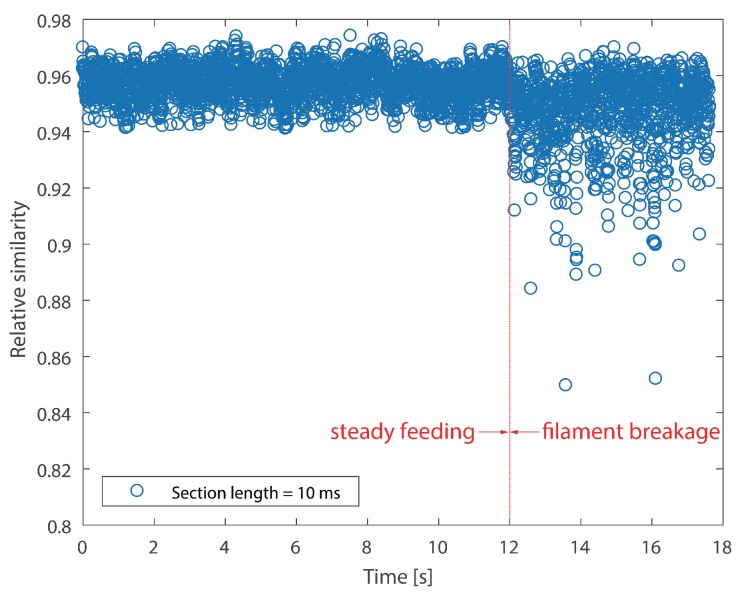
Relative similarity of AE signal for Test #2 (section length = 10 ms).

It cannot be neglected from Figure 8 and Figure 9 that there are several positive values after the break point. They were caused by the unsteady fluctuations after breakage. Such a phenomenon should be eliminated. In order to extract features that could precisely represent the variations in the probability distribution, a random time section was selected from steady feeding AE signals and designated as the reference. The relative similarity was calculated using the proposed framework. The bin width was fixed as 0.001 volts, because the quantization resolution of the DAQ was 18 bit and the voltage input range was ±10 v. A single bin width contained only one possible voltage value. Results are described in Figure 10 and Figure 11.

As can be seen in Figure 10, two stages, i.e., steady feeding and filament breakage, are clearly identified. The relative similarity values are higher and closer to one at steady feeding stage, because AE signals at this stage are similar to the reference. The relative similarity values decrease markedly with fluctuations after breakage. This is due to the differences between the breakage AE signals and the reference. In contrast to Figure 8 and Figure 10, the boundaries of the two stages are much more clear.

The effectiveness of instantaneous skewness and relative similarity were also quantified by the Coefficient of Variation (CV). CV is a standardized measure of the dispersion of a probability distribution and is defined as the ratio of the standard deviation δ to the mean μ,

(13) $$CV=\frac{\delta }{\mu }.$$

A CV of zero suggests that all values are the same with no variability, while wider scatters should have larger CV values. The results of the instantaneous skewness and relative similarity are displayed in Table 3.

It is noticed that CV values of the instantaneous skewness are markedly higher than the relative similarity in Table 3. This suggests that the instantaneous skewness experienced more significant fluctuations than the relative similarity, as can be seen from the above four graphs. This indicates that the skewness is more sensitive to the breakage. However, the boundary of instantaneous skewness is not clear enough. Overall, the results suggest that the relative similarity is better than the instantaneous skewness in filament breakage detection.

### 4.2. Discussion

The proposed methods involve limited pre-defined parameters. However, the length of the time section could influence the results of both the instantaneous skewness and the relative similarity. As a consequence, it should be cautiously determined.

**Figure 12:**
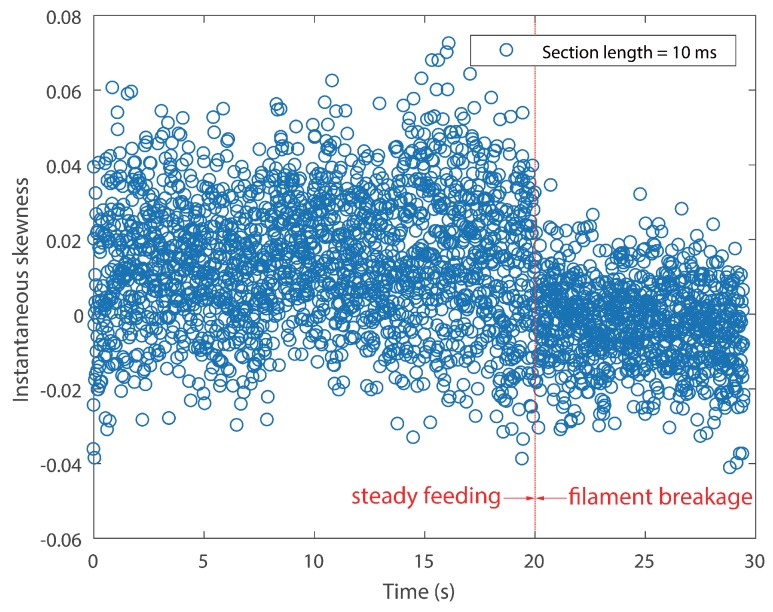
Instantaneous skewness of AE signal for Test #1 (section length = 10 ms).

**Figure 13:**
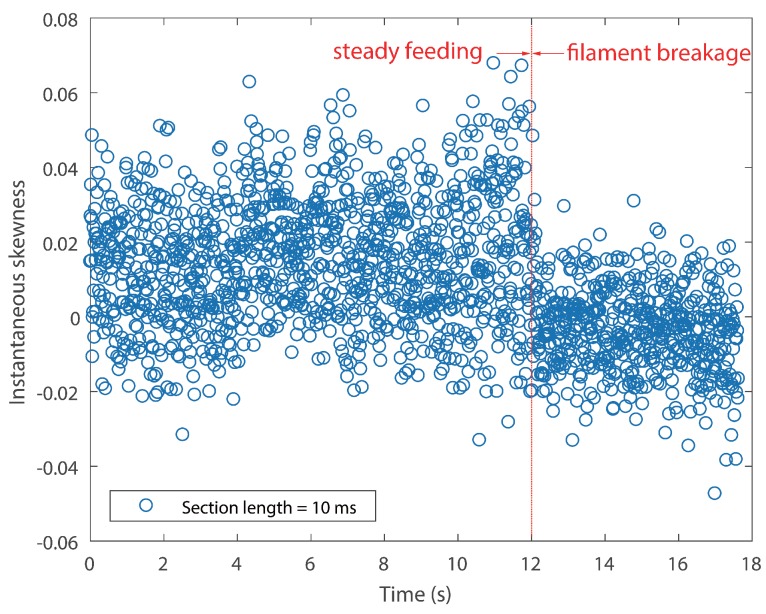
Instantaneous skewness of AE signal for Test #2 (section length = 10 ms).

The temporal resolution and computation time of instantaneous skewness are dependent on the section length time. In general, longer length brings worse temporal resolution, but less computation time. Therefore, the length should be determined based on the monitoring object. In this work, the filament feeding rate is relatively slow. As a consequence, a longer time section was selected. The results of a section length of 10 ms for both tests are displayed in Figure 12 and Figure 13.

As expected, the scatter densities are higher in Figure 12 and Figure 13 and give rise to more fluctuations than Figure 8 and Figure 9. The CV values of both tests rise with the increase of temporal resolution, as can be seen in Table 4. Both negative skew and positive skew occupy the scatter. Although the two stages could be identified visually, their boundaries are still vague.

**Figure 14:**
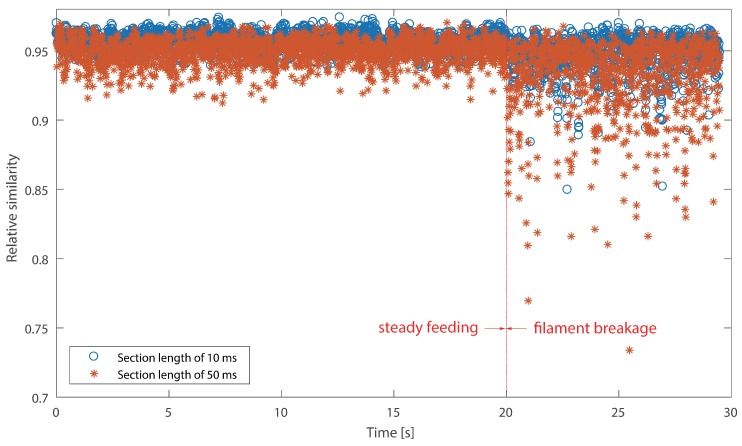
Relative similarity results using different time sections for Test #1.

As regards relative similarity, the section length time is also linked to the temporal resolution of the identification result. A longer section length will give rise to more computation time for the calculation of the probability distribution, while a shorter section length will cause a similar consequence since it increases the total amount of time sections. Two different section length times, i.e., 10 ms and 50 ms, were used to calculate the relative similarity. Results on Test #1 are presented in Figure 14. According to the graph, it is clear that different lengths exerted slightly different similarity values. The relative similarity is slightly lower at a section length of 50 ms. However, the break point can be identified from both scatters. Furthermore, Table 5 proclaims that a longer section produces slightly higher CV, which suggests that the relative similarity experienced more fluctuations with the increase of section length, as we expected. The results of Test #2 have similar trends as Test #1, so they are not displayed in this section.

## 5. Conclusions

The aim of the present study was to identify filament breakage features through the acoustic emission technique. Filament breakage is a common failure in FDM processes. It could cause several malfunctions such as nozzle clogging, geometrical misalignments or manufacturing failure. To address this issue, firstly, the present work analyzed the mechanism of filament breakage. A critical feed rate was obtained, which is dependent on the process parameters. Secondly, the feasibility of identifying filament breakage using the AE technique was depicted. Based on the FDM process and AE technique, it is deduced that AE signals after breakage should have a different probability distribution, which was further realized using two quantified indicators, i.e., instantaneous skewness and relative similarity. The frameworks for calculating both indicators are thoroughly described. Afterwards, the proposed methods were validated through several FDM tests. Results indicated that the instantaneous skewness could be used as a preliminary indicator for filament breakage. However, it is not good enough to represent the malfunction. In contrast, filament breakage could be clearly identified via relative similarity. The breakage states could be separated from the steady feeding states. The influences of section length were discussed. It is found that the increase of section length could increase the relative similarity. Nevertheless, it did not add difficulties for filament breakage identification.

The results of the present work could provide a potential approach for in situ process monitoring of the FDM process. For future work, monitoring other process malfunctions, such as nozzle clogging and manufacturing failure using the AE technique in the FDM process will be further studied. Building a closed-loop FDM process monitoring system based on the acoustic emission technique will be another interesting work.

## Figures and Tables

**Table 1:** Parameters of FDM machines.

Model	Materials	Layer Thickness (mm)	Size Capacity (W × H × L (mm))	Nozzle Diameter
Stratasys uPrint	ABS	0.254, 0.330	203 × 203 × 152	0.5 mm
JG Aurora	ABS, PLA	0.100∼0.300	180 × 180 × 280	0.5 mm

**Table 2:** Critical feed rate parameters.

**Parameters**	*E*	*d*	$D_{2}$	η	*l*	*L*	*W*	*H*
**Values**	1.50 GPa	1.75 mm	0.50 mm	155 Pa·s	3.50 mm	8.00 mm	0.50 mm	0.20 mm

**Table 3:** CV values for instantaneous skewness and relative similarity.

Parameters	Test #1	Test #2
Instantaneous skewness	1.25	1.23
Relative similarity	0.014	0.011

**Table 4:** CV values of instantaneous skewness for different time sections.

Parameters	Length of 10 ms	Length of 50 ms
Test #1	2.07	1.25
Test #2	1.93	1.23

**Table 5:** CV values of relative similarity for different time sections.

Parameters	Length of 10 ms	Length of 50 ms
Relative similarity	0.014	0.020

## Abbreviations

The following abbreviations are used in this manuscript:

FDM	Fused Deposition Modeling
AE	Acoustic Emission
AM	Additive Manufacturing
FFF	Fused Filament Fabrication
ABS	Acrylonitrile Butadiene Styrene
PLA	Polylactic Acid
PP	Polypropylene
